# Intraperitoneal implantation of life-long telemetry transmitters in otariids

**DOI:** 10.1186/1746-6148-4-51

**Published:** 2008-12-10

**Authors:** Markus Horning, Martin Haulena, Pamela A Tuomi, Jo-Ann E Mellish

**Affiliations:** 1 Department of Fisheries and Wildlife, Marine Mammal Institute, Oregon State University, Newport, OR 97365, USA; 2 The Marine Mammal Center, 1065 Fort Cronkhite, Sausalito, CA 94965, USA; 3 Alaska Sea Life Center, 301 Railway Ave, Seward, AK 99664, USA; 4 School of Fisheries and Ocean Sciences, University of Alaska Fairbanks, AK 99775, USA; 5 Vancouver Aquarium, PO Box 3232, Vancouver, BC V6B3X8, Canada

## Abstract

**Background:**

Pinnipeds, including many endangered and declining species, are inaccessible and difficult to monitor for extended periods using externally attached telemetry devices that are shed during the annual molt. Archival satellite transmitters were implanted intraperitoneally into four rehabilitated California sea lions (*Zalophus californianus*) and 15 wild juvenile Steller sea lions (*Eumetopias jubatus*) to determine the viability of this surgical technique for the deployment of long-term telemetry devices in otariids. The life history transmitters record information throughout the life of the host and transmit data to orbiting satellites after extrusion following death of the host.

**Results:**

Surgeries were performed under isoflurane anesthesia and single (n = 4) or dual (n = 15) transmitters were inserted into the ventrocaudal abdominal cavity via an 8.5 to 12 cm incision along the ventral midline between the umbilicus and pubic symphysis or preputial opening. Surgeries lasted 90 minutes (SD = 8) for the 19 sea lions. All animals recovered well and were released into the wild after extended monitoring periods from 27 to 69 days at two captive animal facilities. Minimum post-implant survival was determined via post-release tracking using externally attached satellite transmitters or via opportunistic re-sighting for mean durations of 73.7 days (SE = 9.0, *Z. californianus*) and 223.6 days (SE = 71.5, *E. jubatus*).

**Conclusion:**

The low morbidity and zero mortality encountered during captive observation and post-release tracking periods confirm the viability of this surgical technique for the implantation of long-term telemetry devices in otariids.

## Background

The determination of age-specific survival rates and long-term survival (beyond five years) of individual animals is crucial for the effective monitoring and management of wild pinniped populations [1,2] and for the assessment of success and impact of stranded animal rehabilitation programs [3]. In addition, multi-year data on diving and foraging behavior will facilitate the testing of specific hypotheses related to seasonal, ontogenetic, ocean-climate related and anthropogenic changes in declining or endangered species [1,2,4-6]. Classic approaches to the monitoring of wild pinnipeds, including external or internal telemetry transmitters, do not readily permit the collection of this type of data. External transmitters typically do not survive the annual molt. Implanted telemetry devices circumvent external attachment limitations and have been successfully used on a wide range of marine endotherms. However, reception range and area coverage from VHF implants is smaller than for external devices. Transmitting life span is limited to two to three years. While external tags frequently use satellite links for data recovery, this is not currently a technically viable option for reasonably sized, fully implanted devices. Mark re-sight studies based on hot-iron branding or numbered flipper tags require very large sample sizes [7]. The cost and effort required to collect re-sight information at sufficient sites and frequency further constrain such approaches. In addition, such data do not allow a direct distinction between dispersal and mortality and do not provide information on causes of mortality.

To address these issues, a new telemetry device was developed in collaboration with Wildlife Computers, Inc. (Redmond, WA) for long-term monitoring of pinnipeds: the satellite-linked life history transmitter (LHX tag), described in detail by Horning & Hill [8]. Life history transmitters are implanted into the peritoneal cavity of sea lions under sterile surgical conditions. The tags monitor sensors and store data in memory but do not attempt to transmit until after the animal has died. Once a tag is extruded from a deceased animal, the positively buoyant tag will transmit all previously stored data, including time and date of death. Through the absence of any transmissions until after extrusion, tag life is extended beyond ten years. By linking to the ARGOS system aboard NOAA polar orbiting satellites, coverage for tag monitoring is global, resulting in a spatially and temporally unconstrained re-sight effort.

One complication resulting from the reliance on end-of-life transmissions for all data recovery is the inability to transmit periodic 'alive' signals that are used in conventional mortality transmitters to verify proper transmitter operations. This necessitates accurately determining tag failure rates. To determine failure rates, dual LHX tags are deployed in implanted animals. In addition to increasing data recovery likelihood, the ratio of single to dual tag returns will allow the estimation of tag failure rates. The deployment of LHX tags will permit the application of new experimental designs that are based on long-term, longitudinal monitoring of individual animals and on the direct comparison of survivors to non-survivors.

Here we describe the first surgical, intraperitoneal device implantation in pinnipeds. We implanted LHX transmitters in four California sea lions (*Zalophus californianus*) and 15 Steller sea lions (*Eumetopias jubatus*), and report on postoperative monitoring, and minimum confirmed postoperative survival for periods up to 895 days.

## Methods

### Study area

This study was conducted at two locations in California and Alaska, between May 2004 and June 2008. California sea lions (*Zalophus californianus*) were implanted at The Marine Mammal Center (TMMC), a stranded-animal rehabilitation center in Sausalito, California. The sea lions were released in the Marin Headlands near TMMC and near the Moss Landing Marine Laboratory South of Santa Cruz. Juvenile Steller sea lions (*Eumetopias jubatus*) were captured near Glacier Island in Prince William Sound, Alaska, and transported to the Alaska Sea Life Center (ASLC) at Seward, located at the Northern end of Resurrection Bay, Alaska. The animals were subsequently released into Resurrection Bay near Seward.

### Telemetry transmitters

The life history transmitters [8] are 122 mm long, positively buoyant cylinders with an outer diameter of 42 mm, hemispherical ends, and a mass of 115 g. The tags are coated in Epo-Tek^® ^302-3M medical grade epoxy (Epoxy Technology, Billerica, MA) certified to USP Class VI standards for biocompatibility with implantation in non-cured and polymerized states. This material prevents adhesion to connective tissue or omentum and, therefore, decreases the likelihood of transmitter ingestion by predators of sea lions. Tags were sterilized in ethylene oxide gas (Anprolene^®^, Andersen Products Inc., Haw River, NC). The LHX transmitters were programmed to transmit abdominal temperatures for postoperative monitoring, at hourly intervals and for periods of up to two weeks following implantation, to a nearby handheld UHF receiver, on all animals except CSL6053, CSL6160, TJ22, TJ23 (Additional file 1).

### Subjects, surgical preparations, anesthesia

Four stranded California sea lions (CSL) were selected after completion of rehabilitation procedures at TMMC (Additional file 1). One animal (CSL6053) had been treated for a gastrointestinal foreign body (fish hook, see [9]), and three had been treated for domoic acid toxicity (see [10]). Fifteen wild juvenile Steller sea lions (SSL) were captured for research purposes in Prince William Sound, Alaska (Additional file 1) and transported to a quarantined research facility at the ASLC [11]. The first two animals of each species received single transmitter implants; all remaining animals received dual implants. This study was conducted in compliance with all applicable research ethics and animal welfare regulations. The research protocol was approved by the Institutional Animal Care and Use Committees of the ASLC (AUPs #02-015 & #03-007) and TMMC (AUP #02/04) and by the Office of Protected Resources (U. S. National Marine Fisheries Service) under research permits #1034-1685 and #881-1668.

**Figure 1:**
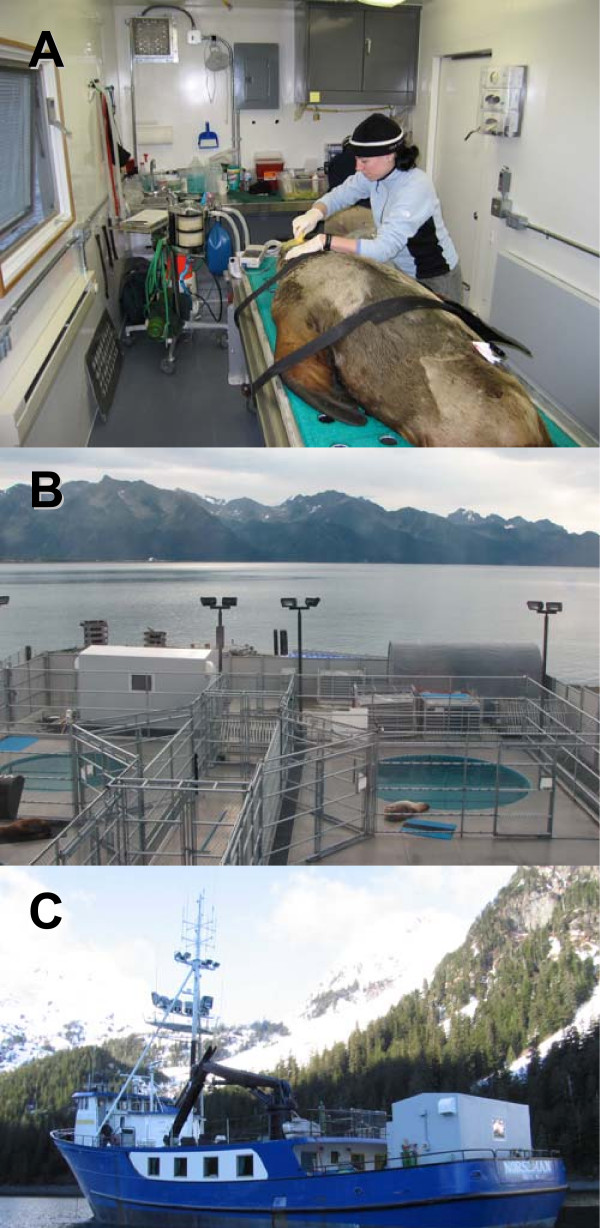
**The portable surgical unit used for Steller sea lion procedures**. (A) Inside view. The unit has one window and standard door, one 2 m wide door to facilitate animal transportation, a stainless steel sink and counter top, and is outfitted with hook-ups for electricity and running water, heating and ventilation, folding work benches, surgical cold light source, a wheeled, adjustable height stainless steel surgical table, and a portable anesthesia machine. (B) The surgical unit located at the quarantined holding facility of the Alaska Sea Life Center, and on the rear deck of the research support vessel MV Norseman (C).

CSL surgeries were performed at TMMC in a hospital building that included a sterile surgery room designed for pinnipeds. All SSL surgeries were performed in a specially designed portable surgical unit (Reiff Manufacturing, Walla Walla, WA) at the ASLC (Figure 1). The containerized surgical unit measures 2.8 × 6.0 m and includes power, heating, running water, ventilation, and surgical equipment. The unit can be transported on a flat bed truck and can be lifted onto the working deck of research vessels. All sea lions were fasted for approximately 12 hours prior to surgery.

California sea lion anesthesia was induced with a mixture of medetomidine (Domitor^®^, Pfizer Animal Health, Exton, PA, 70 μg/kg) and zolazepam-tiletamine (Telazol^®^, Fort Dodge Animal Health, Fort Dodge, IA, 1 mg/kg) by intramuscular (IM) injection delivered by hand or via dart [12]. Steller sea lions were masked with 5% isoflurane (AErrane^®^, Fort Dodge Animal Health) in 100% oxygen after they voluntarily entered squeeze cages [13] for induction of anesthesia [14]. All animals were intubated using appropriately sized, cuffed endotracheal tubes (10 – 16 mm diameter) and anesthesia maintained with 1% to 3% isoflurane in 100% oxygen delivered via a semi-closed, partially rebreathing circuit. Depth of anesthesia was assessed based on respiratory rate and tidal volume, response to stimuli, palpebral reflex, capillary refill time, and jaw and muscle tone. Respiratory and heart rate, oxygen saturation of hemoglobin (SpO_2_), end-tidal carbon dioxide (EtCO_2_) and deep rectal or esophageal body temperature were monitored during anesthetic procedures. Intermittent positive pressure ventilation was provided when indicated (i.e. decreased respiratory rate, decreased SpO_2 _or increased EtCO_2_). Animals that showed continuous irregularities in respiratory parameters were provided with mechanically assisted ventilation through a volume-regulated ventilator (Model 2000, Hallowell EMC, Pittsfield, MA) at a rate of six to ten breaths per minute and a volume of approximately 15 – 20 ml/kg/breath. Hypothermia was prevented with recirculating water or electric heating pads, thermal insulating pads covering the surgical table, warm water bottles placed under the flippers, and/or a Bair Hugger^® ^Model 505 forced warm air blanket (Arizant, Eden Prairie, MN). At the end of surgical procedures, radiographs were taken for the first three CSL prior to recovery from gas anesthesia to determine exact location of implants inside the peritoneal cavity.

Anesthesia was partially reversed in animals induced with medetomidine-zolazepam-tiletamine using atipamezole (Antisedan^®^, Pfizer Animal Health, Exton, PA, 200 μg/kg) IM at the end of the procedure [12]. After surgery, isoflurane and oxygen were discontinued, and animals were maintained on room air via endotracheal tube during recovery. Animals were extubated once jaw tone and the swallowing reflex had returned and then were allowed to recover in dry anesthetic pens (CSL) or transport cages (SSL) for one to five hours before being given unrestricted access to seawater in pens with pools.

All sea lions were given the non-steroidal anti-inflammatory agent flunixin meglumine (Banamine^®^, Schering-Plough Animal Health Corporation, Summit, New Jersey) at 1 mg/kg IM for postoperative analgesia.

### Postoperative monitoring

Following implant procedures, all animals were closely monitored at each facility for periods ranging from 41 to 69 days (CSL) and 27 to 55 days (SSL). For a separate study, fecal samples were collected from animals prior to and after procedures to determine corticosteroid levels [15]. SSL received periodic health screenings (including the collection of venous blood samples for hematology and clinical chemistry) at scheduled intervals prior to release for other research purposes [11]. Radiographs were taken in one California sea lion (CSL6039) prior to release on day 43 post-surgery and in two Steller sea lions (TJ22, TJ23) on days 28 and 14 post-surgery, respectively, to confirm mobility of the implants.

Prior to release, all animals were marked with plastic flipper tags or by using hot iron branding (Additional file 1), and they received external, satellite-linked telemetry transmitters to monitor post-release behavior. These devices (SDR-T16 or SPLASH tags, Wildlife Computers, Inc., Redmond, WA; SRDL tags, SMRU Ltd., St. Andrews, UK) were glued to the dorsal pelage using two-component epoxy [16].

## Results

### Surgical procedure

The animals were positioned securely on a stainless steel surgical table in dorsal recumbence. An area approximately 14 cm long × 8 cm wide was clipped on the ventral abdominal midline caudal to the umbilicus and cranial to the pubic symphysis and preputial opening. The surgical site was cleaned and disinfected using routine surgical preparation with povidone iodine scrub, 70% isopropyl alcohol, and povidone iodine solution. A nonporous, sterile, fenestrated drape was placed over the surgical site and secured with towel clamps.

A longitudinal skin incision of approximately 8.5 to 12 cm in length was made along the ventral midline. The linea alba was exposed using sharp-blunt dissection through blubber and subcutaneous fat layers. The linea alba was lifted with forceps to permit penetration of the abdominal wall with a single stab incision using a scalpel blade. The linea alba was then sharp dissected with scissors, avoiding the peritoneum and viscera, to a length sufficient to pass the transmitter body (approximately 8–12 cm). The peritoneum was visualized, incised with scissors, and the opening expanded manually. The transmitters were removed from the sterilization peel pouches and rinsed in sterile saline solution to facilitate insertion. The abdominal wall was grasped on either side of the incision with Allis forceps or hand held retractors and lifted up and laterally while inserting the transmitters through the incision into the ventrocaudal abdominal cavity. Bleeding was controlled when necessary with hemostatic forceps and ligatures of 2-0 PDS II absorbable monofilament suture (Ethicon^®^, Inc., Somerville, NJ). The surgical incision was closed in multiple layers using size 1 or 0 PDS II sutures. If possible, the peritoneum was closed in a simple continuous pattern to contain intra-abdominal fat and viscera and ease closure of the body wall. Internal abdominal oblique muscle, linea alba, and external abdominal oblique muscle were incorporated in a simple interrupted or interrupted cruciate pattern to close the body wall. Subcutaneous fat was closed in a simple continuous pattern. Intradermal fat (blubber) was closed in a continuous mattress pattern to appose thick blubber layers. A continuous subcuticular pattern was used to appose skin layers without exposing a knot. Finally, an interrupted cruciate pattern was used on the skin to provide additional support to the skin closure. On the first two procedures in CSL, surgical stainless steel staples were used to close the skin layer. Staples were removed prior to release of the animals. Surgical skin glue was used to secure the skin incision in two SSL.

**Figure 2:**
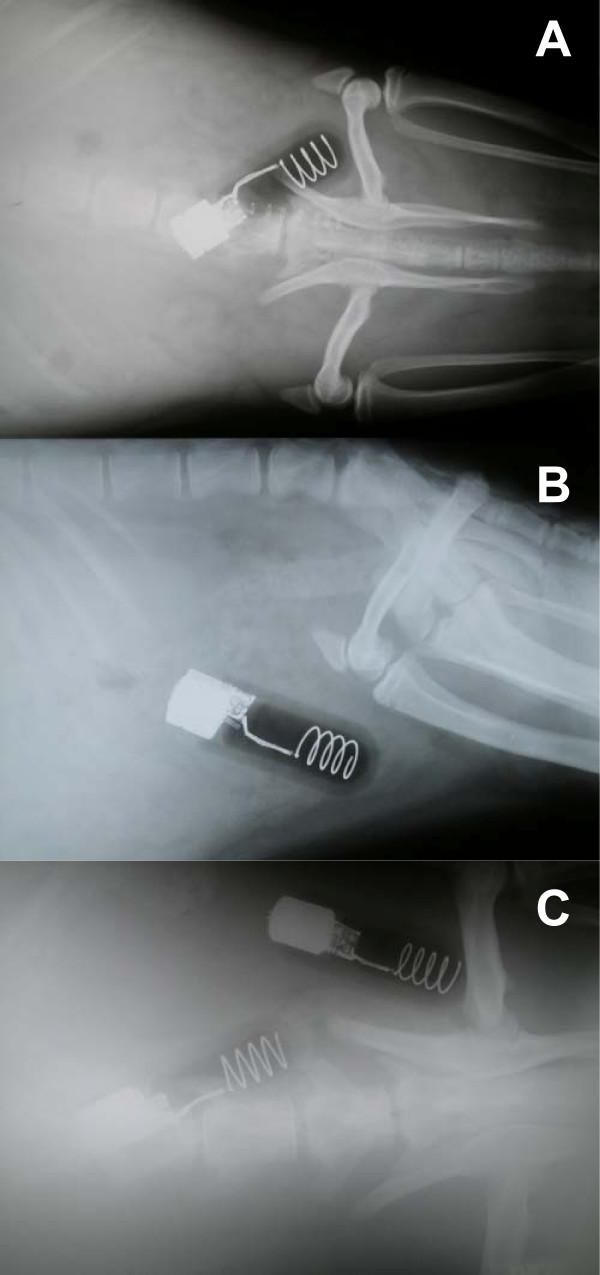
**Post-surgery radiographs of California sea lions with implanted life history transmitters**. (A) Dorsoventral view of single transmitter in animal CSL6018, a 66 kg female – the tag has a size of approximately three vertebrae. (B) Lateral view of single transmitter in CSL6018. (C) Dorsoventral view of dual transmitters in animal CSL6053, a 195 kg male. The tags have a size of approximately two vertebrae.

Mean surgical time was 90 minutes (SD = 8) and mean anesthetic time (from induction to recovery) was 125 minutes (SD = 13) for the 19 sea lions. Figure 2A, B demonstrates the relative size of the LHX implant in the smallest of the four CSL, a female (CSL6018). The single transmitter corresponds to approximately three vertebrae in length. With a mass of only 66 kg, this animal was about half the size of the juvenile Steller sea lions for which the LHX tags were primarily developed. Figure 2C demonstrates the relative size of the implants in a much larger animal, CSL6053 (195 kg), comparable in size to juveniles of the target species (SSL). In this animal, the tags correspond to approximately two vertebrae in length.

### Postoperative monitoring

During the immediate post-procedure recovery period (1–5 hrs), the animals were mildly to moderately lethargic and rested in sternal recumbence. Once given access to pools, the animals went into the water immediately. During the first 18 hours after surgery, signs of some abdominal discomfort associated with the incision site were noted in all animals. This was characterized by the animal choosing to lie almost exclusively in lateral recumbence or by keeping the abdominal midline elevated by folding the posterior flippers under the abdomen if lying in a sternal position. However, the animals were not observed to lick or bite at the incision site or to rub it with nose or mouth. The animals spent a considerable amount of time in the water but also repeatedly entered and exited the water with both patterns reflecting normal, pre-surgery behavior. After 18 hours, signs of discomfort (abdominal midline elevation) gradually lessened, and none were noticed after 24 – 48 hours. Appetite and food intake appeared normal, and defecation and fecal consistency was normal. Immediate postoperative abdominal temperatures were slightly elevated in some animals (38 to 39.2°C) but otherwise were within normal ranges for otariids (36.0 to 37.9°C) with the exception of three instances observed in two CSL. In these two animals temperatures briefly rose above 39°C as a result of further manipulations related to other treatment exams (two events, CSL6018) or antagonistic interactions with other sea lions (one event, CSL6039). All SSL maintained body mass and exhibited no change in body condition as estimated via deuterium oxide dilution as reported elsewhere [17] through the experimental period.

**Figure 3:**
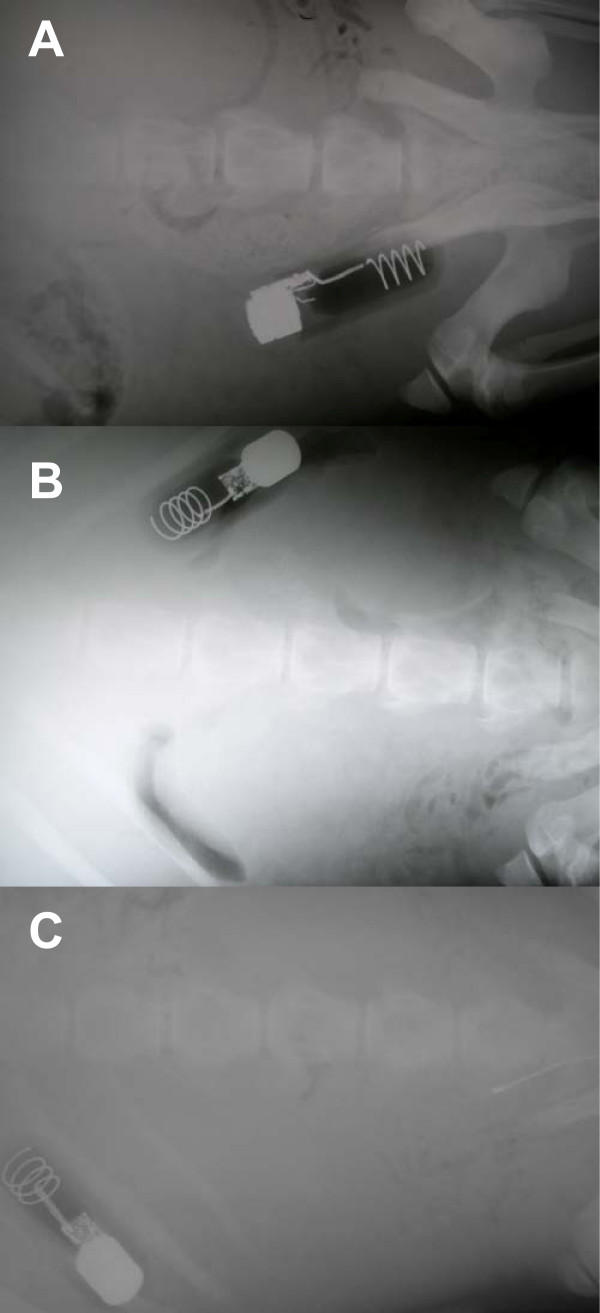
**Radiographs illustrating movement of the free-floating implants**. (A) California sea lion CSL6039 with single implanted life history transmitter on day of surgery, the transmitter is located on the left side of the animal, close to the pelvis. (B) On day 43 post-surgery, the transmitter has moved cranial and to the right side of the abdomen of CSL6039. (C) Steller sea lion TJ22 on day 28 post-surgery. The transmitter is located forward and on the left abdominal side of the animal.

**Figure 4:**
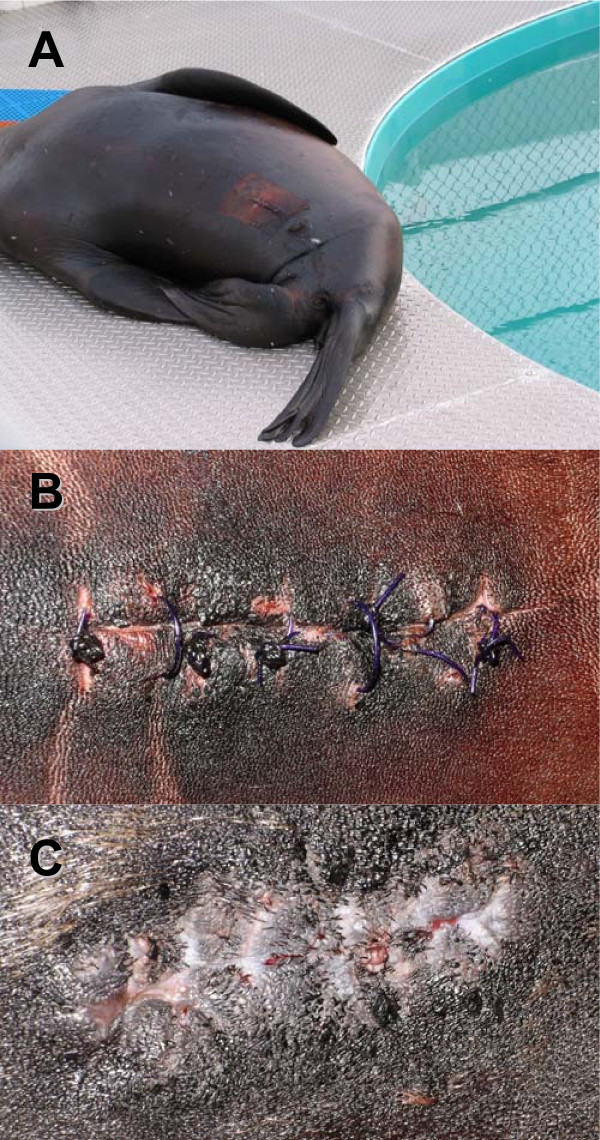
**Incisions at different healing states**. Steller sea lion TJ22 resting poolside 17 days post-surgery (A) and a close up of the healing incision 28 days post-surgery (B): the suture material is not yet dissolved. (C) Incision of California sea lion CSL6039 43 days post-surgery, the suture material has dissolved, the incision shows good granulation.

Figure 3 shows the single LHX tag in CSL6039 immediately after surgery (A), and 43 days after the procedure (B), illustrating the mobility of the transmitters. In TJ22, the single LHX tag had also moved cranial in the animal 28 days after surgery (C). Wound healing progressed very well in all sea lions. Figure 4 shows incisions sites in one SSL after 26 days (A) and one CSL (B) prior to release. All animals showed minimal swelling at the incision site after surgery. A mild clear discharge was noted in two SSL, which may have been associated with skin sutures that were placed too tightly or possibly due to the use of tissue glue preventing drainage. Though surgical steel staples may shorten procedures by up to 15 minutes, they require an additional sedative procedure for removal when compared to absorbable suture on the skin and as a result are not viable for field deployments. No indication of surgical site dehiscence or wound infection was observed as a result of the procedure. No evidence of bacteremia or septicemia was noted in any of the 19 study animals.

All four CSL were deemed releasable based on demonstration of normal behavior, absence of abnormal blood values on CBC and serum chemistry, good body condition, and healed incisions. All 15 SSL were deemed clinically healthy at the time of release based on pre-established release criteria [11]. The four CSL were only monitored for relatively short periods (nine to 47 days) following their release (Additional file 1). This was because release for three animals occurred near the time of annual molt, likely leading to the rapid shedding of external transmitters. These animals were not branded, and no consistent re-sight effort exists in the region. Dive behavior of the four implanted CSL was not compared to control animals since no data on post-release behavior of non-implanted, rehabilitated CSL was available.

Post-release tracking of SSL is reported elsewhere [17], but results revealed diving and ranging behavior comparable to non-implanted control animals [16]. Animals were tracked following their release for 29 to 182 days (Additional file 1). These animals were released in an area with a considerable re-sight effort through research trips (Alaska Department of Fish and Game, Alaska Sea Life Center) and remote video observation systems [18]. Through external tracking devices and re-sight efforts, minimum post-implant survival was confirmed for an average of 73.7 days (SE = 9.0, n = 4 CSL) and 236.6 days (SE = 71.5, n = 15 SSL), respectively (Additional file 1).

## Discussion

Implantable telemetry devices have been used for over 40 years for the study of free-ranging mammals. In a pioneering study, Rawson & Hartline [19] used intraperitoneally implanted radio transmitters for the analysis of movement patterns in deer mice (*Peromyscus maniculatus*). Since the study of Neely & Campbell [20], investigators have attempted to assess the effects of implantation procedures and devices on the behavior and survival of implanted animals. Survival rates have been compared between externally tagged animals and those equipped with either subcutaneous or intraperitoneal telemetry implants. The papers by Folk et al., Neely & Campbell, Smith & Whitney, and MacDonald & Amlaner [21-23] deliver an excellent overview of the early use of implantable telemetry devices in mammals. The predominant problems in early applications relate to issues of relative size as well as packaging and sterility of instruments and procedures. Subsequently, recommendations were made for implanted telemetry devices not to exceed 3–5% of animal body mass [23], although some authors later found no indications of reduced mobility with implants as large as 10% of animal body mass [24]. However, Baumans et al. [25] found significant changes in behavior, feeding, and body mass in laboratory mice that received implants of 12% of body mass, though part of the observed changes were in response to the surgery. Modern implantable telemetry tags typically remain under 1% of body mass, a relative size considered unproblematic.

Initially, some implantation procedures were carried out under "clean but not sterile" conditions [26]. Using appropriate instrument sterilization and sterile surgery techniques, infections from implant procedures have become virtually absent. This has reduced the incidence of post-surgical infections to one in 160 procedures in sea otters (C. Monnett, U. S. Geological Survey, personal communication). In 183 yellow-bellied marmots (*Marmota flaviventris*) with intraperitoneal implants, 30-day survival and growth rates, as well as pregnancy rates and mean litter sizes did not differ from controls [27]. In 53 Eastern wolf (*Canis lycaon*) pups with intraperitoneal implants monitored for up to one year, no postoperative complications, bacteremia or peritonitis were encountered [28]. In those instances where infections occurred in earlier applications (likely as a result of compromised sterility) bacteremia resulted in deaths within the first week after surgery in over 90% of cases [29,30] (C. Monnett, personal communication).

Packaging and specifically the outermost encasing material has an effect on the likelihood of adhesion of intraperitoneal devices to intestines. This adhesion has been reported as responsible for some of the very few observed complications in intraperitoneal implants [31]. Modern inert physiologically compatible resins have resolved this issue [32]. Some researchers prefer to promote connective tissue growth and adhesion, in part to facilitate recovery of implanted devices [33] or to reduce likelihood of interfering with pregnancies and parturition. However, recovery of implanted devices has been feasible with free-floating implants [34]. In addition, studies using free-floating implants have reported fewer mortalities than those using fixed or encapsulating devices [33].

Several studies have reported on the effects of implants on reproduction in aquatic mammals, all using free-floating telemetry implants. Reid et al. [35] studied reproductive effects of intraperitoneal implants in seven adult female North American river otters (*Lontra canadensis*). They observed 12 possible pregnancies resulting in eight litters over one to two reproductive cycles and concluded that the implants did not interfere with reproduction. Hernandez-Divers et al. [36] concluded that intra-abdominal implants did not affect survival or reproductive potential in North American river otters. Fernandez-Moran et al. [37] came to similar conclusion in a study of Eurasian otters (*Lutra lutra*). Nolfo & Hammond [38] intraperitoneally implanted VHF transmitters into 20 adult nutria (*Myocastor coypus*). All 11 females in the study were pregnant. The authors found no evidence of morbidity or infection and concluded that the implants did not interfere with reproduction, though one female aborted her near full-term litter within one day of surgery prior to release, likely as a result of anesthesia. Monnett & Rotterman [32] reported that 17 of 19 implanted adult female sea otters (*Enhydra lutris*) that were deemed pregnant at time of implantation based on abdominal palpation pupped successfully. They were unable to determine whether the remaining two were misclassified as pregnant, aborted prematurely, had stillbirths, or the pups died after birth. All of these studies suggest minimal or no impact of implanted telemetry devices on reproduction in aquatic mammals.

Bodkin et al. [34] surgically implanted two telemetry devices, one VHF transmitter and one time-depth-recorder, into each of 21 sea otters, for the purpose of subsequent recapture and explantation of the archival data logger. The two devices combined were approximately 0.3% and 0.5% of the body mass of male and female otters, respectively. Tags were recovered from 14 animals recaptured after two months and from one dead animal after four years. No reasons for mortality were reported. One pregnant female was implanted and subsequently had a pup.

All early studies comparing subcutaneous to intraperitoneal implantation concluded that the latter was the preferred technique, generating fewer complications than a subcutaneous application [20,39-41]. A more recent study by Lander et al. [42] suggested that subcutaneous implants in harbor seals (*Phoca vitulina*) may be a viable approach, though their results varied. Animals with resin-encased transmitters developed fluid pockets and mucopurulent discharge, whereas wax-coated devices elicited no such response. Nevertheless, the intraperitoneal implantation of telemetry devices into mammals in general (marmots [27]; silver fox [43]; badgers [41]), and aquatic mammals in particular (beavers [44]; muskrat [45]; river otters [30,36,37]; sea otters [32,34]; nutria [38]), has become a reasonably routine procedure. Amongst aquatic mammals, five deaths attributable to implanted devices have occurred in 366 reported procedures since 1983.

In this study single LHX tag mass was less than 0.1% of the mean body mass of 138.5 kg for all implanted sea lions (Additional file 1). However, with a tag diameter of 42 mm and typical blubber depths in Steller sea lions under five cm [46], intraperitoneal implantation was deemed more appropriate than subcutaneous implantation. In addition, this should eliminate the possibility of receiving false mortality information from extruded transmitters and reduce the likelihood of transmitters being ingested by predators. For the same reason a surface coating was chosen that minimizes connective tissue growth and adhesion to the omentum.

Hematology and clinical chemistry data of six SSL (TJ22 – TJ 27) were reported in detail elsewhere [17]. A peak non-significant white cell elevation two weeks post-surgery was noted. Lymphocyte and monocyte values exhibited a significant peak one week post-surgery, with subsequent values below pre-surgery levels. A significant acute phase reaction following implantation of transmitters showed a rise in haptoglobins to a peak at three weeks post-surgery, with subsequent values returning to pre-surgery levels within six weeks. Globulins exhibited a slight though significant elevation three weeks after surgery followed by a gradual decline. Fecal (CSL) and serum (SSL) glucocorticoid levels were also reported elsewhere [15] and indicate limited stress response to surgical procedures, compared to slightly greater responses exhibited by control animals (CSL) to physical restraint without anesthesia.

The comprehensive data from hematology and clinical chemistry in six SSL and from fecal and serum glucocorticoids (in CSL and SSL) showed a return to pre-surgery baseline values in all monitored clinical health parameters by six weeks following implantation with acute phase responses being the most protracted [15,17]. This suggests that the minimum confirmed post-surgery survival of 73 days (mean, range 52 – 95d) for all four *Z. californianus *and 223 days (mean, range 72 – 910d) for all 15 *E. jubatus *is sufficient to establish the survivability of intraperitoneal implantation surgery of telemetry devices. However, long-term effects of intraperitoneal telemetry devices will have to be evaluated within the context of biological applications using such implants. The ultimate measure of such effects will be a comparison of long-term survival rates derived from implants, compared to conventional, mark re-sight techniques [6,7].

## Conclusion

The low morbidity and absence of any mortality encountered in this study during captive observations and post-release monitoring, in conjunction with separately published results on clinical and behavioral effects of implants, illustrate the survivability of intraperitoneal implantation surgery for the deployment of long-term telemetry devices on pinnipeds. Although animals were monitored for extended periods under highly controlled conditions, observed behavior and recovery progress did not suggest any benefits in restricting animal movement or access to seawater beyond the immediate post-surgery anesthetic recovery period.

This technique should be applicable to field deployments, in particular since surgeries could be carried out in the same environment used for the Steller sea lions at the quarantined ASLC facility (Figure 1B). Shipboard inhalant gas anesthesia has been used successfully shortly after capture on well over 100 wild juvenile Steller sea lions for the application of external telemetry devices or biological sampling (J. Mellish, University of Alaska Fairbanks, unpublished data). Implant surgeries could be conducted inside of the mobile surgical unit on board a research vessel (Figure 1C), and animals could be released back into the water after five to ten hours (or about twice the postoperative recovery period reported here) in a suitable confinement area.

## Authors' contributions

MHorning: conceived, designed and implemented the study, assisted in implantation surgeries, participated in postoperative monitoring, conducted post-release monitoring, analysis and interpretation of data, and drafted the manuscript. JEM: designed and implemented the study, assisted in implantation surgeries, participated in postoperative and post-release monitoring, analysis and interpretation of data, and helped to draft the manuscript. MHaulena and PAT developed the implantation surgery protocol, carried out implantation surgeries and postoperative monitoring, and helped to draft the manuscript. All authors read and approved the final manuscript.

## Supplementary Material

Additional file 1 **Summary deployment data for California sea lions and Steller sea lions implanted with LHX transmitters**. The table lists vital data, deployment details and minimum confirmed post-surgery survival for all 19 experimental animals. Click here for file

## References

[B1] Hooker SK, Biuw M, McConnell BJ, Miller PJO, Sparling CE (2007). Bio-logging science: Logging and relaying physical and biological data using animal-attached tags. Deep-Sea Res II.

[B2] Cooke SJ (2008). Biotelemetry and biologging in endangered species research and animal conservation: relevance to regional, national, and IUCN Red List threat assessments. Endang Species Res.

[B3] Moore MG, Early G, Touhey K, Barco S, Gulland F, Wells R (2007). Rehabilitation and release of marine mammals in the United States: Risks and Benefits. Mar Mamm Sci.

[B4] Springer AM, Estes JA, van Vliet GB, Williams TM, Doak DF, Danner EM, Forney KA, Pfister B (2003). Sequential megafaunal collapse in the North Pacific Ocean: An ongoing legacy of industrial whaling?. Proc Natl Acad Sci USA.

[B5] Williams TM, Estes JA, Doak DF, Springer AM (2004). Killer Appetites: assessing the role of predators in ecological communities. Ecol.

[B6] Cooke SJ, Hinch SG, Wikelski M, Andrews RD, Kuchel LJ, Wolcott TG, Butler PJ (2004). Biotelemetry: a mechanistic approach to ecology. Trends Ecol Evol.

[B7] Gerrodette T (1987). A Power Analysis for Detecting Trends. Ecol.

[B8] Horning M, Hill RD (2005). Designing an archival satellite transmitter for life-long deployments on oceanic vertebrates: The Life History Transmitter. IEEE J Ocean Eng.

[B9] Goldstein T, Johnson SP, Phillips AV, Hanni KD, Fauquier DA, Gulland FMD (1999). Human-related injuries observed in live-stranded pinnipeds along the central California coast 1986–1998. Aq Mamm.

[B10] Gulland FMD, Haulena M, Fauquier D, Langlois G, Lander ME, Zabka T, Duerr R (2002). Domoic acid toxicity in California sea lions (*Zalophus californianus*): clinical signs, treatment and survival. Vet Rec.

[B11] Mellish JE, Calkins DG, Christen DR, Horning M, Rea LD, Atkinson SK (2006). Temporary Captivity as a Research Tool: Comprehensive Study of Wild Pinnipeds Under Controlled Conditions. Aq Mamm.

[B12] Haulena M, Gulland FMD (2001). Use of medetomidine-zolazepam-tiletamine with and without atipamezole reversal to immobilize captive California sea lions. J Wildl Dis.

[B13] Christen D, Moundalexis E, Mellish J, Dailer J, Dusage S, Down H, Hartman L (2007). The use of atypically applied operant conditioning methods to manage, care for, and study free-ranging juvenile Steller sea lions in captivity. IMATA Soundings.

[B14] Haulena M, West G, Heard D, Caulkett N (2007). Otariid seals. Zoo Animal and Wildlife Immobilization and Anesthesia.

[B15] Petrauskas L, Atkinson S, Gulland F, Mellish J, Horning M (2008). Monitoring glucocorticoid response to rehabilitation and research procedures in California and Steller sea lions. J Exp Zool Part A Ecol Genet Physiol.

[B16] Thomton JD, Mellish JE, Hennen DR, Horning M (2008). Juvenile Steller sea lion foraging behavior following temporary captivity. Endang Species Res.

[B17] Mellish J, Thomton J, Horning M (2007). Physiological and behavioral response to intra-abdominal transmitter implantation in Steller sea lions. J Exp Mar Biol Ecol.

[B18] Maniscalco J, Parker P, Atkinson S (2006). Interseasonal and interannual measures of maternal care among individual Steller sea lions (*Eumetopias jubatus*). J Mammal.

[B19] Rawson KS, Hartline PH (1964). Telemetry of homing behavior by the deermouse, *Peromyscus*. Science.

[B20] Neely RD, Campbell RW (1973). A system for gathering small mammal mortality data. US Forest Service Research Paper.

[B21] Folk GW, Essler WO, Folk MA (1971). The abdominal cavity for transport of instruments. Fed Proc.

[B22] Smith HR, Whitney GD, Long FM (1977). Intraperitoneal transmitter implants – their biological feasibility for studying small mammals. Proc 1st Intl Conf Wildl Biotelem.

[B23] MacDonald DW, Amlaner CJ, Amlaner CJ, MacDonald DW (1980). A practical guide to radio tracking. A handbook on biotelemetry and radio tracking.

[B24] Koehler DK, Reynolds TD, Anderson SH (1987). Radio-transmitter implants in 4 species of small mammals. J Wildl Manag.

[B25] Baumans V, Bouwknecht JA, Boere H, Kramer K, van Lith HA, Weerd HA van de, van Herck H (2001). Intra-abdominal transmitter implantation in mice: effects on behaviour and body weight. Anim Welf.

[B26] Eagle TC, Choromanskinorris J, Kuechle VB (1984). Implanting radio transmitters in mink and Franklin ground-squirrels. Wildl Soc Bull.

[B27] Van Vuren D (1989). Effects of intraperitoneal transmitter implants on yellow-bellied marmots. J Wildl Manag.

[B28] Crawshaw GJ, Mills KJ, Mosley CM, Patterson BR (2007). Field implantation of intraperitoneal radiotransmitters in eastern wolf (*Canis lycaon*) pups using inhalation anesthesia with sevoflurane. J Wildl Dis.

[B29] Williams TD, Siniff DB (1983). Surgical implantation of radio telemetry devices in the sea otter. J Am Vet Med Assoc.

[B30] Johnson SA, Berkley KA (1999). Restoring river otters in Indiana. Wildl Soc Bull.

[B31] Guynn DC, Davis JR, Von Recum AF (1987). Pathological potential of intraperitoneal transmitter implants in beavers. J Wildl Manag.

[B32] Monnett C, Rotterman LM (2000). Survival rates of sea otter pups in Alaska and California. Mar Mamm Sci.

[B33] Fuglei E, Mercer JB, Arnemo JM (2002). Surgical implantation of radio transmitters in arctic foxes (*Alopex lagopus*) on Svalbard, Norway. J Zoo Wildl Med.

[B34] Bodkin JL, Esslinger GG, Monson DH (2004). Foraging depths of sea otters and implications to coastal communities. Mar Mamm Sci.

[B35] Reid DG, Melquist WE, Woolington JD, Noll JM (1986). Reproductive effects of intraperitoneal transmitter implants in river otters. J Wildl Manag.

[B36] Hernandez-Divers SM, Kollias GV, Abou-Madi N, Hartup BK (2001). Surgical technique for intra-abdominal radiotransmitter placement in North American river otters (*Lontra canadensis*). J Zoo Wildl Med.

[B37] Fernandez-Moran J, Saavedra D, Manteca-Vilanova X (2002). Reintroduction of the Eurasian otter (*Lutra lutra*) in Northeastern Spain: trapping, handling, and medical management. J Zoo Wildl Med.

[B38] Nolfo LE, Hammond EE (2006). A Novel Method for Capturing and Implanting Radiotransmitters in Nutria. Wildl Soc Bull.

[B39] Philo ML, Follman EH (1981). Field surgical techniques for implanting temperature sensitive radio transmitters in grizzly bears. J Wildl Manag.

[B40] Garshelis DL, Siniff DB (1983). Evaluation of radio-transmitter attachment for sea otters. Wildl Soc Bull.

[B41] Agren EO, Nordenberg L, Mörner T (2000). Surgical implantation of radiotelemetry transmitters in European badgers (*Meles meles*). J Zoo Wildl Med.

[B42] Lander ME, Haulena M, Gulland FMD (2005). Implantation of subcutaneous radio transmitters in the harbor seal (*Phoca vitulina*). Mar Mamm Sci.

[B43] Bakken M, Moe RO, Smith AJ, Selle GME (1999). Effects of environmental stressors on deep body temperature and activity levels in silver fox vixens (*Vulpes vulpes*). Appl Anim Behav Sci.

[B44] Wheatley M (1997). A new surgical technique for implanting radio transmitters in beavers, *Castor canadensis*. Can Field-Nat.

[B45] Lacki MJ, Smith PN, Peneston WT, Vogt DF (1989). Use of methoxyflurane to surgically implant transmitters in muskrats. J Wildl Manag.

[B46] Mellish JE, Horning M, York AE (2007). Seasonal and spatial blubber-depth changes in captive harbor seals (*Phoca vitulina*) and Steller's sea lions (*Eumetopias jubatus*). J Mammal.

[B47] King JC, Gelatt TS, Pitcher KW, Pendleton GW (2007). A field-based method for estimating age in free-ranging Steller sea lions (*Eumetopias jubatus*) less than twenty-four months of age. Mar Mamm Sci.

[B48] Mellish JE, Hennen DR, Thomton JD, Petrauskas LR, Atkinson SK, Calkins DC (2007). Permanent marking in an endangered species: physiological response to hot branding in Steller sea lions (*Eumetopias jubatus*). Wildl Res.

