# Supplementary material for: Intraperitoneal implantation of life-long telemetry transmitters in otariids
Source: BMC Vet Res. 2008 Dec 10;4:51. doi: 10.1186/1746-6148-4-51 (PMC2621145; doi:10.1186/1746-6148-4-51)
Supplement: Additional file 1 — Summary deployment data for California sea lions and Steller sea lions implanted with LHX transmitters. The table lists vital data, deployment details and minimum confirmed post-surgery survival for all 19 experimental animals. [file 1746-6148-4-51-S1.doc]

**Table 1:** Summary deployment data for California sea lions and Steller sea lions implanted with LHX transmitters.

| **Animal no.** | **Species ^a^** | **Age ^a^** | **Sex** | **Body  mass ^b^**  **(kg)** | **Number**  **of**  **LHX**  **tags** | **Surgery**  **date** | **Release**  **date** | **External**  **marking ^c^** | **Days**  **tracked ^d^** | **Min. days confirmed post-**  **operative**  **survival ^e^** |
| --- | --- | --- | --- | --- | --- | --- | --- | --- | --- | --- |
| CSL6018 | CSL | 4-5 yrs | F | 66 | single | 18 May 2004 | 29 Jun 2004 | Flipper tag | 28 | 69 (T) |
| CSL6039 | CSL | 5-6 yrs | M | 118 | single | 25 May 2004 | 08 Jul 2004 | Flipper tag | 9 | 52 (T) |
| CSL6053 | CSL | 8-10 yrs | M | 195 | dual | 7 Jul 2004 | 16 Sep 2004 | Flipper tag | 10 | 79 (T) |
| CSL6160 | CSL | 6-7 yrs | M | 140 | dual | 25 Aug 2004 | 13 Oct 2004 | Flipper tag | 47 | 95 (T) |
| TJ22 | SSL | 17 mo | F | 119 | single | 27 Sep 2005 | 22 Nov 2005 | Brand | 125 | 181 (T) |
| TJ23 | SSL | 17 mo | M | 109 | single | 11 Oct 2005 | 22 Nov 2005 | Brand | 72 | 114 (T) |
| TJ24 | SSL | 22 mo | M | 153 | dual | 16 Mar 2006 | 17 Apr 2006 | Brand | 70 | 910 (BR) |
| TJ25 | SSL | 22 mo | M | 152 | dual | 16 Mar 2006 | 17 Apr 2006 | Flipper tag | 70 | 195 (BR) |
| TJ26 | SSL | 22 mo | M | 161 | dual | 15 Mar 2006 | 17 Apr 2006 | Brand | 70 | 462 (BR) |
| TJ27 | SSL | 22 mo | M | 167 | dual | 15 Mar 2006 | 17 Apr 2006 | Brand | 70 | 103 (T) |
| TJ32 | SSL | 16 mo | M | 160 | dual | 13 Sep 2007 | 10 Oct 2007 | Brand | 136 | 165 (T) |
| TJ33 | SSL | 16 mo | M | 134 | dual | 13 Sep 2007 | 10 Oct 2007 | Brand | 124 | 151 (T) |
| TJ34 | SSL | 16 mo | M | 102 | dual | 12 Sep 2007 | 09 Oct 2007 | Brand | 182 | 287 (BR) |
| TJ35 | SSL | 16 mo | M | 149 | dual | 12 Sep 2007 | 09 Oct 2007 | Brand | 122 | 149 (T) |
| TJ36 | SSL | 16 mo | M | 99 | dual | 12 Sep 2007 | 09 Oct 2007 | Brand | 143 | 170 (T) |
| TJ38 | SSL | 22 mo | M | 190 | dual | 1 Apr 2008 | 29 Apr 2008 | Brand | 29 | 230 (BR) |
| TJ39 | SSL | 22 mo | M | 152 | dual | 1 Apr 2008 | 29 Apr 2008 | Brand | 44 | 72 (T) |
| TJ40 | SSL | 22 mo | F | 105 | dual | 2 Apr 2008 | 29 Apr 2008 | Brand | 52 | 79 (T) |
| TJ41 | SSL | 22 mo | M | 160 | dual | 2 Apr 2008 | 29 Apr 2008 | Brand | 60 | 87 (T) |

^a^ California sea lion (CSL) age was estimated based on visual appearance and body mass, Steller sea lion (SSL) age based on tooth eruption patterns [47] and adjusted to peak pupping dates.

^b^ On date of procedure.

^c^ All animals were marked by either plastic flipper tags placed near the axillary region or by hot-iron branding [48].

^d^ Animals were tracked after release via externally-attached satellite-linked transmitters (see *Methods*).

^e^ Minimum confirmed post-operative survival based on either reception of dive behavior data from external satellite-linked data transmitters (T) or confirmed sightings of brands / tags (BR) by trained research teams, or via remote video monitoring systems [18].
